# Burnout and job satisfaction of nursing staff in a South African acute mental health setting

**DOI:** 10.4102/sajpsychiatry.v26i0.1454

**Published:** 2020-07-27

**Authors:** Anthea Payne, Liezl Koen, Daniel J.H. Niehaus, Inge-Marli Smit

**Affiliations:** 1 Department of Psychiatry, Faculty of Medicine and Health Sciences, Stellenbosch University, Cape Town, South Africa

**Keywords:** psychiatric nurses, burnout, job satisfaction, mental healthcare, South Africa

## Abstract

**Background:**

Psychiatric nurses constitute a fundamental part of the mental health care system in South Africa. However, high levels of burnout and job dissatisfaction among nursing staff have been associated with reduced empathy and quality of care, and poor service delivery. Stikland Psychiatric Hospital is a state psychiatric hospital situated in Belville and provides all levels of psychiatric care to a large part of the Cape metro region. To our knowledge, no previous studies have examined burnout and job satisfaction among nurses in this setting.

**Aim:**

We assessed the relationship between burnout and job satisfaction among the nursing staff.

**Setting:**

The study was conducted at Stikland Psychiatric Hospital, Cape Town, South Africa.

**Methods:**

This cross-sectional study used the Copenhagen Burnout Inventory and an established job satisfaction questionnaire to assess burnout and job satisfaction among 127 staff members associated with psychiatric nursing.

**Results:**

In this population comprising mostly female (83.5%) nurses, scores for personal, work-related and client-related burnout were relatively high, but job satisfaction was also high. Higher levels of burnout were significantly associated (Pearson’s linear correlation, *r* = -0.077, *p* < 0.01) with lower levels of job satisfaction. There were no significant associations between burnout or job satisfaction and gender, rank or years of experience.

**Conclusions:**

If mental health service delivery is to be optimised, supportive or preventative processes should be implemented to reduce the prevalence of burnout in psychiatric nurses. This study adds to the scarce local knowledge and provides information that can be used to inform the development of supportive strategies for psychiatric nursing staff in South Africa.

## Introduction

There is a high prevalence of burnout and job dissatisfaction among nursing professionals globally,^1,2^ and in low- to middle-income countries (LMICs) in particular.^3^ Burnout, defined by Schaufeli and Greenglass as ‘a state of physical, emotional and mental exhaustion that results from long-term involvement in work situations that are emotionally demanding’,^4^ has been associated with reduced quality of care and poor service delivery.

It has also been shown to increase the rate of absenteeism among nursing staff members.^5^ Job satisfaction, defined as a pleasurable or positive emotional state resulting from the appraisal of one’s job experiences,^6^ has often been shown to be correlated with burnout. For example, Rosales et al. reported a statistically significant relationship between burnout and job dissatisfaction in Filipino nurses.^7^ However, this positive correlation is not uniform.

An Australian study showed that haemodialysis nurses experienced high levels of burnout despite favourable work environments and acceptable levels of job satisfaction.^8^

There are several factors that have been shown to contribute to burnout and job dissatisfaction. Professionals below the age of 25, who have less than 5 years of experience and low levels of annual income experience higher levels of burnout.^9^ A systematic review showed that job satisfaction in hospital nurses is closely related to working conditions and the organisational environment, job stress, role conflict and ambiguity, role perception and role content, as well as organisational and professional commitment.^10^

In 2000, Weber and Jaekel-Reinhard addressed the controversies associated with the concept of burnout being recognised as a valid work-related disease rather than an opinion or excuse for poor work performance.^11^ These authors concluded that burnout is at times dismissed as a ‘fashionable trend’ or ‘invention of the media’ because of gaps in our knowledge. Burnout requires interdisciplinary input, and if it is disregarded as a medical concept then we would be dismissing an important dimension of holistic care – the social dimension. The study recommends further research to establish a consensus in definitions and diagnostic criteria as well as research into biological, biochemical and molecular effects of stress. Furthermore, they also recommend epidemiological studies to establish potential causal relationships with psycho-social or psycho-mental stress at work to minimise gaps in knowledge around burnout so that the necessary preventative measures and medical care can be implemented.

The nursing profession is a fundamental component of the health care system in South Africa and represents more than 50% of the total professional human resources.^12^ Some studies have shown that South African nursing staff are at a high risk for burnout and job dissatisfaction.^12^ Their results demonstrated the association with particular factors in the practice environment such as wages, opportunities for advancement, study leave, inadequate staffing and resources and lack of nursing participation in hospital affairs. South Africa is a resource-deficient country, and this can also influence the development of burnout in staff.^13^

Although there are challenges in all specialities of nursing, there are particular difficulties that are specifically associated with mental health settings.^9,14,15,16^ These include the intense nature of nurse–patient interaction and the management of difficult and challenging patient behaviours.^17,18^ Burnout in psychiatric nurses has also been found to be associated with depressive and anxiety symptoms.^19^

The South African Stress and Health (SASH) study, a large-scale population-based study of common mental health disorders published in 2009, showed a lifetime prevalence for any mental health disorder of 30.3%, with the Western Cape having the highest 12-month and lifetime prevalence rates.^20^ This burden contributes to increased workloads of staff because of high patient numbers and resultant increased turnover of admissions in psychiatric hospitals. It is important to note that the SASH study did not include some of the causes of ‘serious’ mental illness, such as schizophrenia and other psychotic disorders, which are responsible for much of the burden of psychiatric admissions. Nevertheless, the SASH study and others have emphasises that there is a growing unmet need for mental health services in South Africa.^20,21^

As nurses are at the forefront of mental health care in South Africa, it is paramount that they are adequately supported. Previous research has recommended that the initiation and implementation of programmes aimed to reduce stress and burnout for health care professionals, postulating that these programmes could increase the well-being and resilience of mental health professionals and, in turn, improve the quality of mental health care.^9^ While some data exist on burnout in South African nursing staff, no study has specifically investigated the relationship between burnout and job satisfaction in a psychiatric nursing setting. Therefore, the aim of this study is to determine the prevalence of levels of burnout and degree of job satisfaction for the current nursing staff at Stikland State Psychiatric Hospital and to establish whether these experiences are associated with certain demographic variables.

## Methods

### Study design

This cross-sectional study measured burnout and job satisfaction of permanent nursing staff working at Stikland Psychiatric Hospital, South Africa. The study was conducted between November and December 2017.

### Study setting

Stikland Hospital is a state psychiatric hospital situated in Bellville, South Africa. The facility provides all levels of psychiatric care to a part of the Tygerberg East metro region as well as the Westcoast or Winelands rural region. This includes providing general care to adults as well as specialised geriatric and addiction services.

### Study population

All nurses (*N* = 221) employed by Stikland Psychiatric Hospital on a permanent basis were eligible to participate in the study. These included professional nurses (*N* = 120), staff nurses (enrolled nurses, *N* = 24) and nursing assistants (*N* = 76). Nursing staff employed on a contract basis and students were excluded. The full details of the study were explained to the ward operational managers who informed nurses of the study. They were then approached and invited to participate by the primary investigator. A total of 132 nurses participated (60% response rate), but data of five participants were excluded because of incomplete demographic questionnaires.

Therefore, the data obtained from 127 nurses were included in the study.

### Data collection

Envelopes containing the informed consent document as well as the demographic questionnaire, Copenhagen Burnout Inventory (CBI) and Job Satisfaction Survey (JSS) were distributed to all the wards on the premises of Stikland Psychiatric Hospital. Nurses filled out these self-administered questionnaires and deposited their responses into sealed collection boxes that were stored by the operational managers in a secure area. The collected data were then entered into an Excel spreadsheet by the primary investigator.

### Measures

The demographic information questionnaire collected data related to age, gender, rank, ward category, years of experience, duration of employment at Stikland Psychiatric Hospital and full-time or part-time employment.

The CBI^22^ and the JSS^23^ were used to measure burnout and job satisfaction, respectively.

The CBI is a 19-item questionnaire that measures three burnout sub-dimensions (personal, work-related and client-related burnout). The personal burnout sub-dimension of the CBI measures ‘the degree of physical and psychological fatigue and exhaustion experienced by the person’. The work-related sub-dimension measures ‘the degree of physical and psychological fatigue and exhaustion that is perceived by the person as related to his or her work’. Client-related burnout is defined as ‘the degree of physical and psychological fatigue and exhaustion that is perceived by the person as related to his or her work with clients’.^22^ The CBI has been used in both general and psychiatric nursing studies and has also been validated in a general South African occupational group.^16,24,25^ All items are rated on a five-point Likert scale ranging from 1 (never or almost never) to 5 (always). Responses for each question are given scores between 0 and 100. Scores are summed for each sub-dimension and higher scores indicate more burnout.

Oshagbemi^23^ originally developed the JSS to measure the job satisfaction of university teachers.

The JSS has not been validated internationally or locally. It consists of four questions that are rated on a five-point Likert scale.^23^ The first option scores one point, and subsequent responses are given an increasing number of points as per numerical progression. Total job satisfaction scores are categorised as follows: 20–17, very satisfied; 16–13, satisfied; 12, indifferent; 11–8, dissatisfied and 7–4, very dissatisfied.

### Data analysis

Continuous variables were summarised as mean and standard deviation (SD), while categorical variables were summarised as counts and percentages. Valid percentages were used to take into account the fact that some participants did not fully complete the CBI or JSS. Because of the small sample size, scores for the three burnout sub-dimensions were summed to provide a single (total) burnout score. Linear regression (Pearson’s product-moment coefficient) was used to show the relationship between total burnout scores and job satisfaction scores, as well as between burnout and years of experience. Independent *t*-tests were used to test the differences in CBI and job satisfaction scores between gender, while analysis of variance (ANOVA) was used to test the difference of CBI and JSS between ranks. Statistical significance was evaluated at the 5% significance level (*p* < 0.05), and all analyses were performed using SPSS (IBM, version 24).

### Ethical considerations

The study was approved by the Health Research Ethics Committee (HREC) of Stellenbosch University as well as the Stikland Facility Management and Ethics Committee (reference number: 1116). Anonymity of participants was maintained throughout, and the consent document stated that implied consent would be indicated by completion of the questionnaires.

## Findings

The demographic characteristics of the 127 psychiatric nurses who participated in this study are presented in Table 1. Most participants (83.5%) were female, and more than half (59.1%) were ranked as professional nurses. The mean ± SD age of participants was 43.46 ± 11.53 years (range: 23–63 years). Mean years of total nursing experience (including psychiatric nursing) as well as duration of experience at Stikland Psychiatric Hospital were found to be 17.1 years (median: 15.5 years; mode: 5 years; range: < 1 year to 45 years) and 11.5 years (median: 7 years; mode: 2 years; range: < 1 year to 35 years), respectively.

**TABLE 1:** Personal and job demographic characteristics of psychiatric nurses (*n* = 127) recruited from Stikland Psychiatric Hospital.

Variables	Frequency (*n*)	%
**Gender**		
Male	21	16.5
Female	106	83.5
**Rank**		
Professional nurse	75	59.1
Registered nurse	19	15.0
Nursing assistant	33	26.0
**Ward category**?		
Male acute	43	35.2
Female acute	30	24.6
Geriatrics	18	14.8
Therapeutic	21	17.2
Chronic	10	8.2

† , Five participants did not indicate their respective ward category.

**FIGURE 1-A1:**
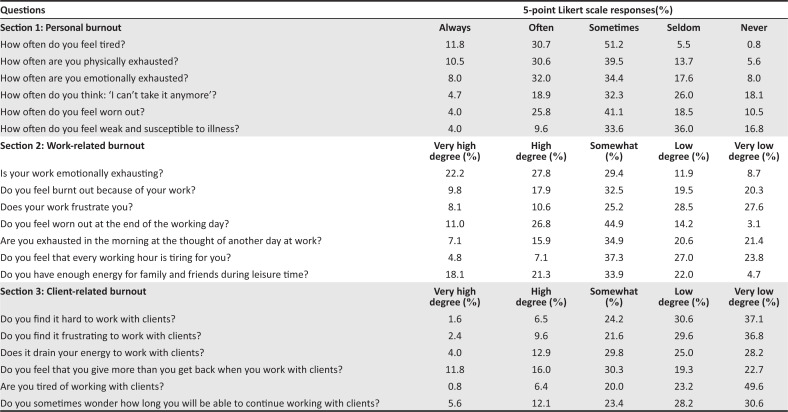
Frequency of responses to the Copenhagen Burnout Inventory (*n* = 127).

The frequencies of individual responses to CBI and JSS are provided in Appendix 1, Figure 1-A1 and Table 1-A1, respectively (supplementary information). The mean ± standard deviation scores for personal, work-related and client-related burnout were 49.2 ± 19.95, 45.0 ± 21.90 and 26.0 ± 19.49, respectively. The total score for job satisfaction was 15.5 ± 2.81 which indicates that psychiatric nurses at Stikland Psychiatric Hospital are satisfied overall. Linear regression showed that a negative correlation exists between burnout and job satisfaction (Pearson’s product-moment correlation, *r* = -0.077, *p* < 0.01), indicating that higher levels of burnout are associated with lower levels of job satisfaction in this population. There were no significant associations between burnout or job satisfaction and gender, rank or years of experience.

## Discussion

This study examined levels of burnout and job satisfaction among psychiatric nurses employed at Stikland Psychiatric Hospital. Overall, results showed that nurses displayed relatively high levels of burnout but were, nevertheless, satisfied with their jobs. Furthermore, higher levels of burnout were associated with lower levels of job satisfaction in this population, but there was no significant relationship between burnout or job satisfaction and selected demographic factors (gender, rank and years of experience).

In our population of psychiatric nurses, the mean job satisfaction score was 15.5 which indicates that these nurses are fairly satisfied with their jobs. A systematic review by Lu et al. reviewed numerous articles and found that there are varying levels of job satisfaction found in nurses in different settings.^10^ The following factors were found to lead to high job satisfaction: degree of cohesion, perception of staff organisation, salaries, opportunities for advancement, co-workers, extrinsic reward, autonomy, communication, administrative, recognition, working conditions, professional practice, organisational support and practices, physical and psychological responses to work as well as patient relationships.^10^ Our study did not particularly explore which factors contributed to high job satisfaction within the population at Stikland Hospital but we recommend further research, perhaps in the form of a qualitative study to further explore this finding.

We found high levels of job satisfaction. The score for client-related burnout was lower in our study (26.0) compared to that (30.9) found by Kristensen et al.^22^ This was somewhat surprising because psychiatric nursing is known to be difficult because of the presenting behaviours of patients who can be aggressive or suicidal.^26^ However, scores for personal (49.2) and work-related (45.0) burnout were higher than scores of 35.9 and 33.0 reported previously.^15^ When comparing personal and work-related burnout, the scale is able to distinguish persons who are fatigued because of non-work factors such as health or family demands. A higher score for personal burnout indicates that personal factors, such as health or family demands, are key factors contributing to burnout among this sample of nurses. This could possibly explain why job satisfaction remains high despite high levels of burnout.

Our results show that higher levels of burnout are significantly associated with lower levels of job satisfaction.

The literature around burnout in South Africa remains scarce. Khamisa et al. also showed that burnout and job satisfaction were negatively correlated in a Johannesburg private nursing population.^27^

Engelbrecht et al. explored predictors and levels of burnout experienced by professional nurses working in Primary Health Care facilities in the Free State, South Africa and found that high levels of burnout were associated with the availability of resources, workload and conflict at work.^28^ When comparing studies in other LMICs, Van Der Doef et al. showed that East African nurses experience greater understaffing, heavier workload, less favourable physical working conditions and less availability and poorer quality of equipment and materials.^29^ Their study revealed that burnout scores showed high levels of emotional exhaustion and depersonalisation in East African nurses. International studies have also showed that the higher the level of job satisfaction, the lower the risk of burnout.^7,26^ However, our study also showed that despite high levels of burnout, job satisfaction remained high. Other South African studies have shown that positive practice environments increase job satisfaction and lower levels of burnout in critical care nurses.^12^ Future research using a qualitative study method would be useful to determine the reasons for high job satisfaction despite high levels of burnout in this population.

The results of this study showed no significant correlations between burnout or job satisfaction and gender or rank. Hayes et al. also found no statistically significant correlations between rank and burnout or job satisfaction, as well as between gender and burnout or job satisfaction.^8^ Chakraborty et al. found no significant correlation between the level of burnout and rank.^16^ A previous meta-analysis conducted using the literature originating from the USA showed that the correlation between burnout and years of experience is inconclusive.^30^ It may be necessary to investigate this potential relationship further, especially within the local context.

One of the limitations of this study was the relatively poor response rate (60%), possibly because we asked nurses to fill out the survey in their own time. Providing protected time to participate may have increased the response rate. This could be a source of bias because we cannot be sure about the levels of burnout and job satisfaction in the other nurses who chose not to participate. The study also had a relatively small sample size, and results are not generalisable to other psychiatric hospitals. In particular, the working environment at Stikland Psychiatric Hospital does not accurately reflect the diversity of mental health service conditions across South Africa.

Furthermore, this study was conducted in a public hospital in South Africa, and the results do not necessarily reflect the private hospital nursing population. The public sector hospitals are faced with additional challenges such as resource limitations and increased patient turnover which could influence the work environment.

Studies using larger samples of participants recruited from multiple sites are encouraged for future research.

Similar studies in other more resource constrained and rural settings in South Africa would increase our understanding regarding the extent of burnout in the local context.

## Conclusion

Despite high levels of burnout in this population of nurses at Stikland Psychiatric Hospital, the staff were still ‘satisfied’ with their jobs. No significant correlations were found between burnout or job satisfaction and gender, rank or years of experience. It is our recommendation that preventative or supportive measures be implemented to reduce the prevalence of burnout in nursing staff.

## Appendix 1: Supplementary information

**TABLE 1-A1:** Frequency of responses to the job satisfaction questionnaire (*n* = 127).

Question	5-point Likert scale responses (%)				
	1	2	3	4	5
**Which one of the following indicates how much of the time you feel satisfied with your job?**	3.2	5.6	24.0	48.8	18.4
1. Never	-	-	-	-	-
2. Seldom	-	-	-	-	-
3. About half of the time	-	-	-	-	-
4. Most of the time	-	-	-	-	-
5. All of the time	-	-	-	-	-
**Which one of the following statements best describes how you feel about your job?**	0.0	4.0	7.2	48.0	40.8
1. I hate it	-	-	-	-	-
2. I dislike it	-	-	-	-	-
3. I’m indifferent to it	-	-	-	-	-
4. I like it	-	-	-	-	-
5. I love it	-	-	-	-	-
**Which one of the following statements best describes how you feel about changing your current job?**	4.8	4.8	13.7	36.3	40.3
1. I would quit this job at once if I could	-	-	-	-	-
2. I would like to change my job soon	-	-	-	-	-
3. I am not sure if I would exchange my present job for a similar one	-	-	-	-	-
4. I am not eager to change my job, but I would do so if I could get a better job	-	-	-	-	-
5. I would not exchange my job for any other	-	-	-	-	-
**Which one of the following statements best describes how you think you compared with other people?**	0.8	5.6	42.7	43.5	7.3
1. No one dislikes his/her job more than I dislike mine	-	-	-	-	-
2. I dislike my job more than most people dislike theirs	-	-	-	-	-
3. I like my job about as well as most people like theirs	-	-	-	-	-
4. I like my job better than most people like theirs	-	-	-	-	-
5. No one likes his/her job better than I like mine	-	-	-	-	-
